# Endoplasmic reticulum-translocation is essential for APOL1 cellular toxicity

**DOI:** 10.1016/j.isci.2021.103717

**Published:** 2021-12-31

**Authors:** Etty Kruzel-Davila, Ira Bavli-Kertselli, Ayala Ofir, Amber M. Cheatham, Revital Shemer, Eid Zaknoun, Sergiy Chornyy, Orly Tabachnikov, Shamara E. Davis, Atanu K. Khatua, Karl Skorecki, Waldemar Popik

**Affiliations:** 1 Department of Nephrology, Rambam Health Care Campus, Haifa, Israel; 2 Departments of Genetics and Developmental Biology and Rappaport Faculty of Medicine and Research Institute, Technion—Israel Institute of Technology, Haifa, Israel; 3 Meharry Medical College, Center for AIDS Health Disparities Research, Department of Microbiology and Immunology, 1005 D. B. Todd Boulevard, Nashville, TN 37028, USA; 4 Department of Internal Medicine, 1005 D. B. Todd Boulevard, Nashville, TN 37028, USA

**Keywords:** Cellular physiology, Cell biology, Functional aspects of cell biology

## Abstract

Two variants at the *APOL1* gene, encoding apolipoprotein L1, account for more than 70% of the increased risk for chronic kidney disease in individuals of African ancestry. While the initiating event for APOL1 risk variant cell injury remains to be clarified, we explored the possibility of blocking APOL1 toxicity at a more upstream level. We demonstrate that deletion of the first six amino acids of exon 4 abrogates APOL1 cytotoxicity by impairing APOL1 translocation to the lumen of ER and splicing of the signal peptide. Likewise, in orthologous systems, APOL1 lethality was partially abrogated in yeast strains and flies with reduced dosage of genes encoding ER translocon proteins. An inhibitor of ER to Golgi trafficking reduced lethality as well. We suggest that targeting the MSALFL sequence or exon 4 skipping may serve as potential therapeutic approaches to mitigate the risk of CKD caused by APOL1 renal risk variants.

## Highlights

• Deletion of the first six amino acids of exon 4 (ΔMSALFL) abrogates APOL1 toxicity
• ΔMSALFL impairs APOL1 translocation to the ER lumen and splicing of signal peptide
• Impaired ER translocation and ER to Golgi trafficking reduced APOL1 toxicity
• MSALFL sequence targeting or exon 4 skipping could yield possible therapy

## Graphical abstract

**Figure:**
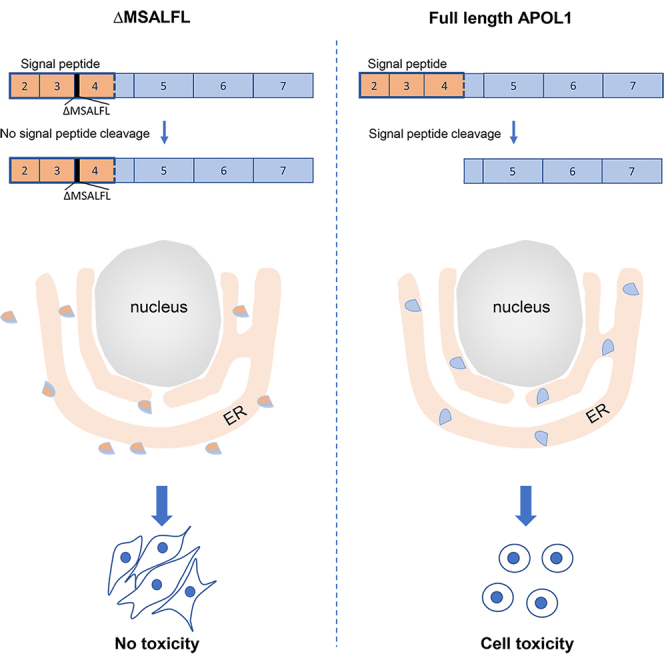

## Introduction

Two derived allelic variants of the *APOL1* gene (encoding Apolipoprotein L1), designated as G1 (encoding S342G and I384M substitutions) and G2 (encoding N388 and Y389 deletions), are causally associated with a markedly increased risk for various forms of non-diabetic chronic kidney disease, in comparison with the ancestral allele G0 (Genovese et al., 2010; Tzur et al., 2010; Beckerman et al., 2017; Fu et al., 2017; Kruzel-Davila et al., 2017a). These mutant alleles are estimated to account for more than 70% of the increased burden of non-diabetic CKD in the African ancestry population (Dummer et al., 2015; Friedman and Pollak, 2016; Kasembeli et al., 2015; Kopp et al., 2011; Kruzel-Davila et al., 2015, 2017b). It is estimated that more than 70 million people worldwide (including approximately 6 million African Americans) have the high-risk *APOL1* genotype comprising two parental risk alleles. Functional APOL1 protein is found only in humans, gorillas, mangeby, mandrill, and baboons (Smith and Malik, 2009; Thomson et al., 2014) and is dispensable for kidney function (Smith and Malik, 2009; Johnstone et al., 2012). Several studies have explored APOL1-mediated cellular injury using *in vitro* and *in vivo* models and suggested a gain-of-function injury mechanism, but a complete picture remains to be elucidated (Beckerman et al., 2017; Fu et al., 2017; Granado et al., 2017; Kruzel-Davila et al., 2017a; Lan et al., 2015a; Ma et al., 2017; Nichols et al., 2015; Olabisi et al., 2015; Thomson et al., 2014; Wen et al., 2018; Lan et al., 2014; Cheng et al., 2015; Heneghan et al., 2015; Lan et al., 2015b; Bruggeman et al., 2016; Bruggeman et al., 2017; O'Toole et al., 2018). Alternative “loss of protection” mechanisms have also been proposed to explain the recessive mode of CKD risk inheritance (Kruzel-Davila et al., 2015). Recent studies using a variety of model systems including yeast, fly, mouse, and human cells in culture, have converged on mechanisms wherein APOL1 interferes with core processes that relate to endosomal trafficking pathways and impaired acidification of the endolysosomal organelles, potassium efflux, and disrupted autophagic flux, with downstream activation of pyroptotic cell death, mitochondrial dysfunction, and ER stress (Beckerman et al., 2017; Fu et al., 2017; Granado et al., 2017; Kruzel-Davila et al., 2017a; Ma et al., 2017; Wen et al., 2018; Olabisi et al., 2015).

The *APOL1* gene is encoded by seven exons that can be differentially spliced, usually by exclusion of exon 2 or 4, to encode three major *APOL1* splice isoforms: A, B, and C (Khatua et al., 2015; Nichols et al., 2015; Smith and Malik, 2009; Cheatham et al., 2018). Isoform A is encoded by exons 1 and 3–7. Exons 2, 3, and 4 contribute to the putative NH2-terminal signal peptide involved in ER targeting, membrane trafficking, and protein secretion. Splice isoform B1 is encoded by exons 1–7, whereas isoform B3 lacks exon 4. Isoform C lacks exons 2 and 4 (Khatua et al., 2015). Human kidney glomerular (podocytes, mesangial, and endothelial) cells and tubular cells stimulated with IFNγ, predominantly express *APOL1* splice transcripts with exon 4 (isoform A) (Cheatham et al., 2018). The G1 and G2 alleles are located in the last and largest exon 7; therefore, all APOL1 splice isoforms can harbor these mutations, if present. However, the function of APOL1 proteins encoded by alternatively spliced *APOL1* isoforms in the absence or presence of G1 or G2 risk alleles is unknown. Thus, toxic APOL1 moieties may vary depending on the transcription and epigenetic factors that govern overall *APOL1* gene expression and splicing variation (Cheatham et al., 2018). Because splicing is itself under the influence of external stimuli, it is conceivable that the transformation from risk to disease may reflect the effects of altered splicing. Cheatham et al. have recently demonstrated that suboptimal *cis*-acting RNA regulatory motifs are responsible for constitutive splicing of exon 4, suggesting that lack of an optimal consensus hnRNP A1 motif in exon 4 may explain the robust inclusion of this exon in *APOL1* transcripts and expression of the signal peptide in major APOL1 protein isoforms (Cheatham et al., 2018). Khatua et al. demonstrated that isoforms B3 and C, which lack exon 4 and the putative signal peptide, are non-toxic compared to isoforms that harbor exon 4 (Khatua et al., 2015). These studies were conducted in experimental model systems in which even G0 is injurious, and the mechanisms of loss of toxicity were not clarified in these studies.

Herein, we demonstrate that deletion of the first six amino acids of exon 4 attenuates the cellular toxicity of APOL1 G0, G1, and G2. These amino acids are essential for translocation to the ER lumen and thereby splicing of the signal peptide. In addition, we demonstrate that ER to Golgi trafficking is important for APOL1 toxicity. Based on the recent findings of Cheatham et al. (Cheatham et al., 2018) and the data presented herein, we suggest that inhibition of exon 4 splicing may serve as a potential therapeutic target to mitigate the risk of chronic kidney disease mediated by APOL1 mutants.

## Results

### Deletion of the first amino acids of exon 4 rescues APOL1 toxicity

HEK 293T cells expressing APOL1 splice isoforms B3 and C that harbor the G1 and G2 mutations demonstrated significantly reduced toxicity compared to the A and B1 isoforms (Figures S1 and S2).

**Figure 1:**
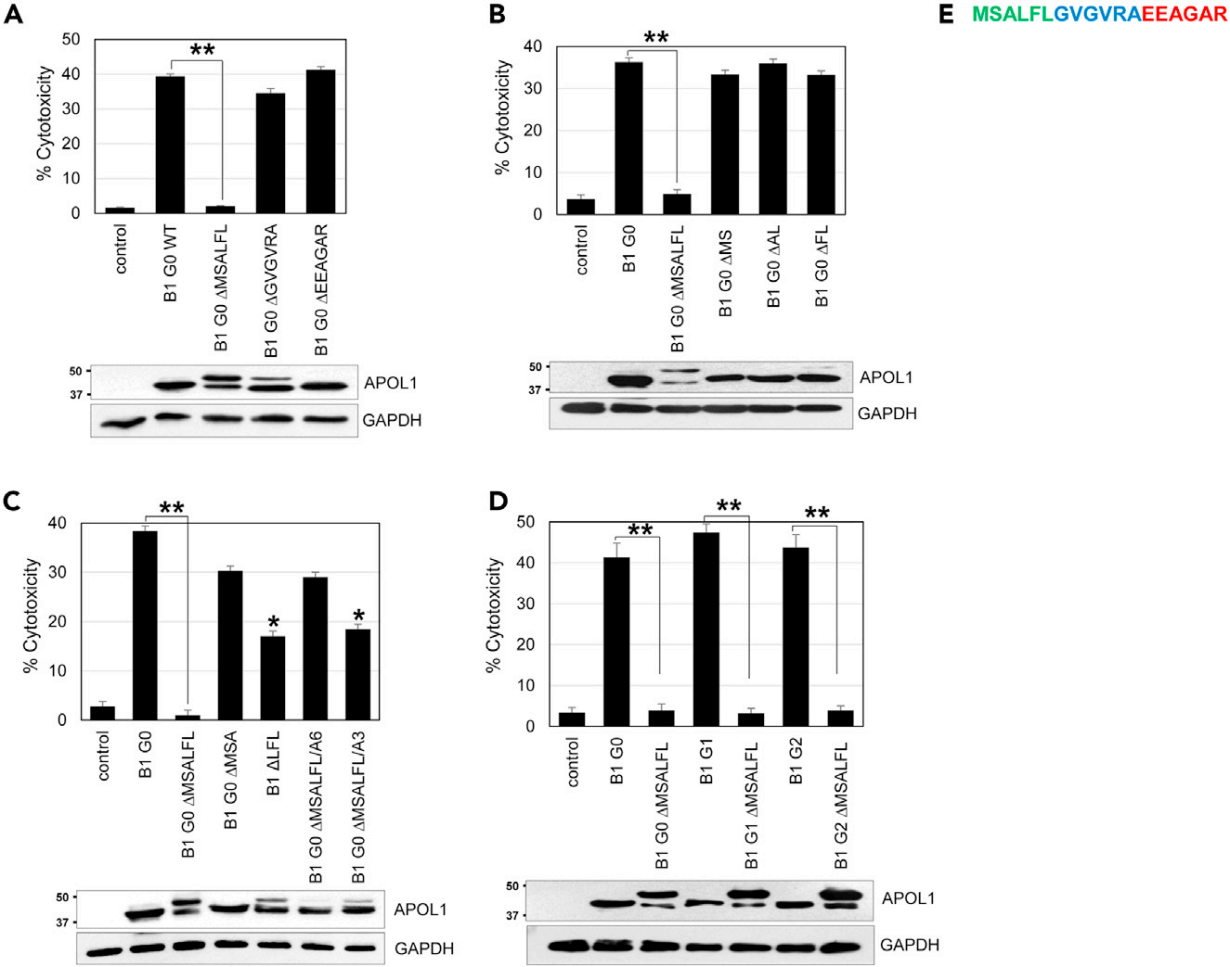
Deletion of the first 6 amino acids of exon 4 inhibits APOL1 toxicity (A) Deletion of the first 6 amino acids (aa) encoded by exon 4 (MSALFL), but not the next 6 aa sequences (GVGVRA and EEAGAR), strongly inhibits APOL1 toxicity in HEK293T cells measured by LDH assay. Values are means ±SD of 3 independent experiments. (B) Deletion of only 2 aa-MS, AL, or FL (of the MSALFL sequence) is not sufficient to reduce APOL1 cytotoxicity. (C) Deletion of the first 3 aa of MSALFL (MSA) or last 3 aa (LFL) partially restores cytotoxicity compared to ΔMSALFL. Values are means ±SD of 3 independent experiments. Substitution of MSALFL with 6 alanine aa (A6) or with 3 alanine aa (A3) partially restores cytotoxicity compared to ΔMSALFL. (D) Deletion of MSALFL is sufficient to reduce APOL1 toxicity independently of G1/G2 mutations. Values are means ±SD of 3 independent experiments. T test for statistical significance was conducted for each pair in panels A–D, ∗P<0.05, ∗∗P<0.001 were considered statistically significant. (E) Amino acid sequence of the *APOL1* exon 4.

Deletion of the first 6 amino acids MSALFL of exon 4 attenuated APOL1 cellular toxicity, whereas deletion of the next 6 amino acids GVGVRA and EEAGAR, respectively, did not (Figure 1A). Likewise, deletion of 2 amino acids from the MSALFL sequence did not attenuate cellular toxicity significantly (Figure 1B). Deletion of only LFL amino acids increased cell viability slightly but to a lesser degree compared to the deletion of MSALFL (Figure 1C). Similarly, substitution of MSALFL with AAAAAA only partially restored cytotoxicity compared to the absent cell cytotoxicity in ΔMSALFL-expressing cells (Figure 1C). Deletion of the MSALFL sequence was also able to mitigate the cellular toxicity of the renal risk variants (G1 and G2) (Figure 1D). Although APOL1 protein levels were comparable, reduced APOL1 toxicity was accompanied by the appearance of a higher molecular weight band, suggesting impairment of protein processing to the mature form. The pattern of two closely spaced protein bands is consistent with the possibility that the signal peptide cleavage is impaired.

### Deletion of MSALFL impairs the splicing of *APOL1* signal peptide

**Figure 2:**
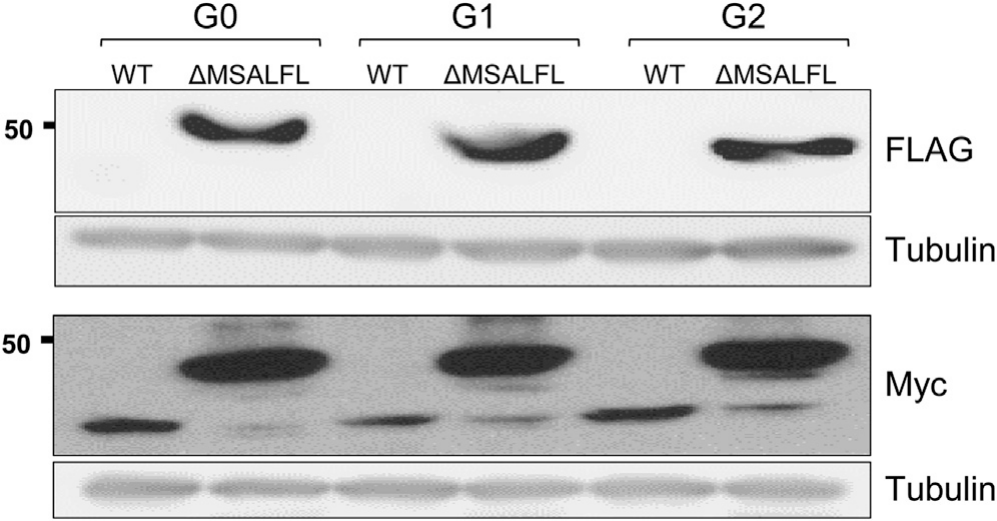
Deletion of MSALFL prevents the signal peptide cleavage *APOL1* constructs that contain a FLAG tag upstream the signal peptide and Myc-tag at the C-terminus were introduced into HEK293 cells. WB with anti-Flag demonstrates that the FLAG tag is intact in the ΔMSALFL but cleaved in the WT form. The MW also fits to a larger protein product that harbors the signal peptide.

In order to explore whether the deletion of these six amino acids affects cleavage of the signal peptide, we used *APOL1* constructs tagged with Flag upstream of the signal peptide and with Myc at the C-terminus. As expected, only the ΔMSALFL variant was detected by anti-Flag antibody (with a higher molecular weight than the WT APOL1), implying that the APOL1 signal peptide was not cleaved, whereas the WT APOL1 as well as the ΔMSALFL APOL1 were detected by anti-Myc (Figure 2). Regardless of APOL1 mutations at the C-terminus, namely G0, G1, and G2, only the ΔMSALFL variants were detected by anti-Flag due to uncleaved signal peptide (Figure 2). These findings favor the formulation that reduced toxicity observed in cells expressing the ΔMSALFL variant is the consequence of aberrant ER translocation leading to signal peptide cleavage impairment.

### The ΔMSALFL pattern of cellular localization is different from the WT isoform

**Figure 3:**
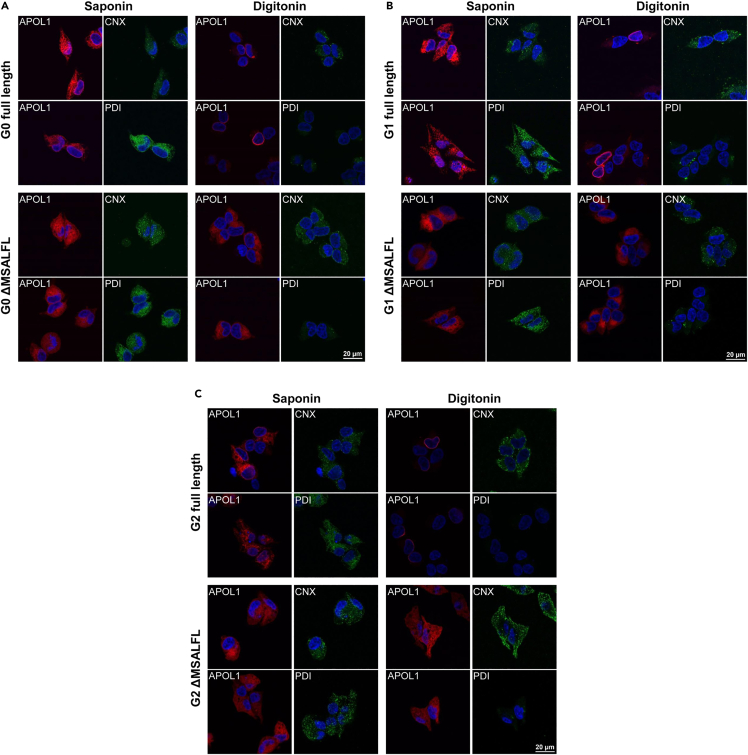
ΔMSALFL constructs localize at the cytoplasmic face of the ER as opposed to the luminal ER localization of full-length APOL1 constructs (A–C) Inducible T-REx 293 cells were induced for APOL1 (flag-APOL1-myc) expression by doxycycline (20 ng/mL) and permeabilized with saponin (left) or digitonin (right). The reticular ER pattern persists in digitonin (permeabilizes plasma membrane only) permeabilized cells expressing the ΔMSALFL constructs (Fig A, B, C – lower panel) but not full-length APOL1 G0, G1, and G2 (Fig A, B, C – upper panel), indicating ΔMSALFL constructs localize at the cytoplasmic face of the ER, while full-length APOL1 is localized inside the ER, thereby not acceciable to staining after digitonin permeabilization as opposed to saponin permeabilization. Calnexin (CNX C-terminal part) and PDI were used as controls for cytoplasmic and luminal ER localization, respectively.

In order to explore the reason for impaired signal peptide cleavage, we investigated whether the trafficking and localization of ΔMSALFL construct is altered. APOL1 variants co-localized with the ER marker calnexin (Figure S3), similar to previous studies that have demonstrated that APOL1 is localized to the ER (Granado et al., 2017; Kruzel-Davila et al., 2017a; Wen et al., 2018; Gupta et al., 2020; Scales et al., 2020). Such ER luminal localization provides an environment that is optimized for protein folding and maturation, including signal peptide cleavage. Therefore, we sought to examine whether the ER translocation of ΔMSALFL constructs are perturbed leading to impaired signal peptide cleavage. Scales et al. have recently demonstrated that APOL1 isoforms A and B1 were localized to the luminal face of the ER and to the cell surface, whereas isoforms C and B3 lacking exon 4 were localized to the cytoplasmic face of the ER and were consequently absent from the cell surface (Scales et al., 2020). As previously shown by some (Khatua et al., 2015) but not all groups (Wakashin et al., 2020), splice isoforms C and B3 are less toxic compared to isoform A and B1 (Figure S2). We examined whether the ΔMSALFL variants localize to the outer (cytoplasmic) face of the ER, by permeabilizing inducible T-REx 293 cells with digitonin, which selectively permeabilizes the plasma membrane, leaving the ER membrane intact. All ΔMSALFL variants exhibited a similar ER staining pattern with digitonin as with saponin which permeabilizes all cell membranes, implying that these variants localize to the cytoplasmic face of the ER, similar to the cytoplasmic detection of calnexin. Full-length APOL1 demonstrates nuclear membrane staining but not ER staining after digitonin permeabilization indicating that these APOL1 variants localize inside the ER and are not accessible after digitonin permeabilization (similar to the PDI control for luminal ER localization) (Figures 3 and S4). Cellular fractionation assays also demonstrated that ΔMSALFL APOL1 was found in the ER as well as cytosol, whereas full-length APOL1 was not found in the cytosolic fraction (Figure S5). These findings suggest that impaired ER translocation and *trans* localization at the ER cytoplasmic face disrupt signal peptide cleavage in the ΔMSALFL constructs, thereby preventing Golgi and plasma membrane trafficking and reducing cellular toxicity.

### *Saccharomyces cerevisiae* expressing ΔMSALFL APOL1 are less sensitive to APOL1 toxicity and demonstrate a different pattern of APOL1 localization

**Figure 4:**
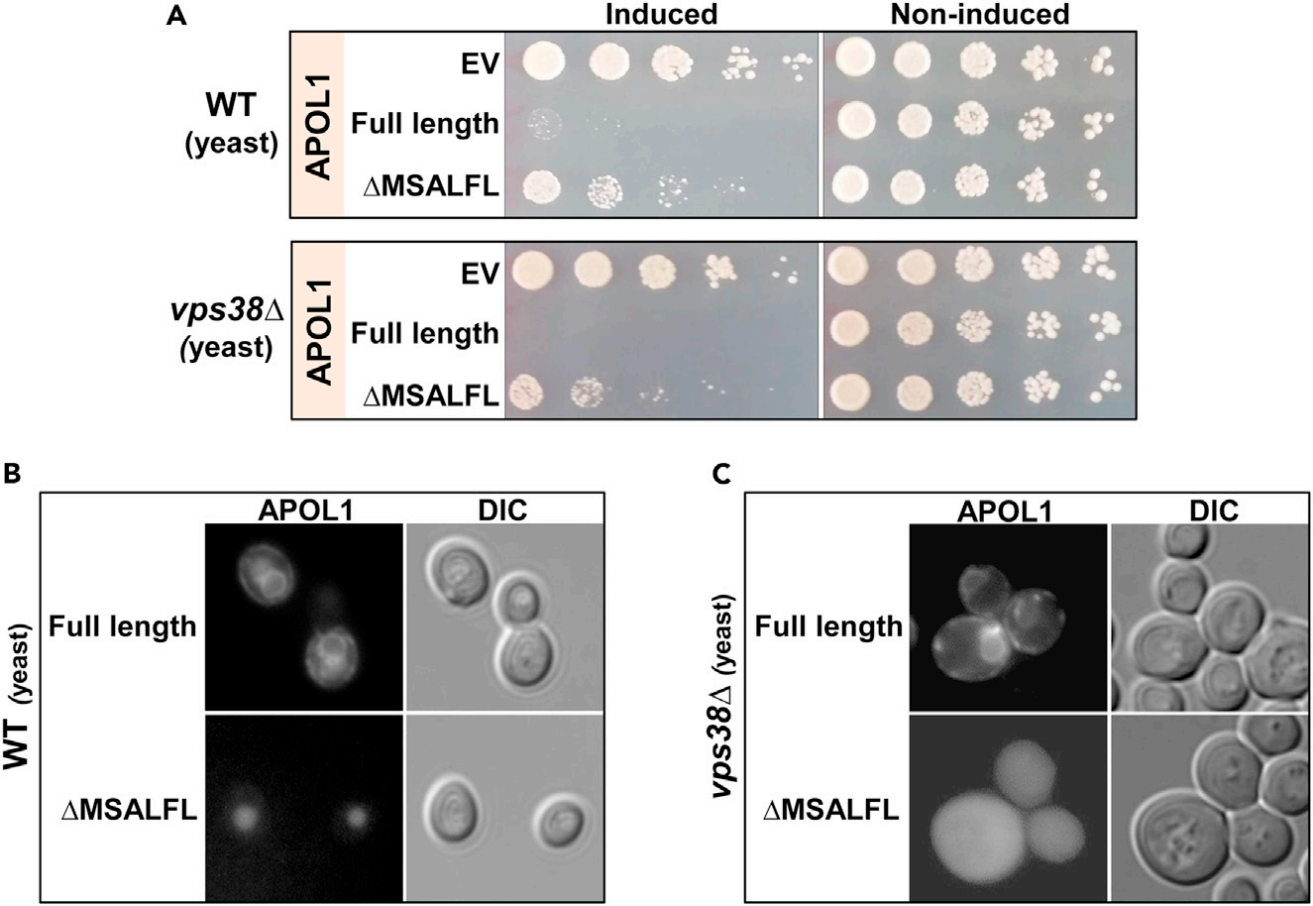
Deletion of MSALFL in yeast attenuates APOL1 toxicity and affects its localization Plasmids containing the human *APOL1* full-length and ΔMSALFL fused to mCherry at the C-terminal, or empty vector (EV) were transformed into WT and *vps38Δ* yeast strains (A) Drop titration assay demonstrates that ΔMSALFL APOL1 mutant expression is less toxic in WT and *vps38Δ* strains than full-length APOL1. (B) Immunofluorescence of APOL1-mCherry demonstrates that full-length APOL1 is expressed in the ER and vacuole; however, the ΔMSALFL is expressed only in the vacuole. (C) Immunofluorescence of APOL1-mCherry in *vps38Δ* demonstrates that full-length APOL1 is diverted from the vacuole to the ER and plasma membrane while the ΔMSALFL APOL1 has a homogeneous distribution in the cytoplasm and as expected was not translocated to the ER, plasma membrane, or vacuole.

We previously reported that the yeast *Saccharomyces cerevisiae* is an informative model to explore the mechanism of APOL1 renal risk variant toxicity (Kruzel-Davila et al., 2017a), and showed that APOL1 was localized to the ER and vacuole (Kruzel-Davila et al., 2017a). Close juxtaposition of the ER to the plasma membrane did not allow us to determine a separate plasma membrane localization. Yeast expressing the ΔMSALFL APOL1 were less sensitive to cell injury than full-length APOL1, recapitulating the phenomenon observed in HEK cells (Figure 4A). As expected, ΔMSALFL translocation to the ER and plasma membrane were impaired compared to full-length APOL1, while vacuole localization was preserved (Figure 4B). The cytoplasm to vacuole targeting (Cvt) pathway that does not involve ER translocation may mediate direct ΔMSALFL APOL1 to the vacuole in yeast (Lynch-Day and Klionsky, 2010). Surprisingly, even the *vps38*Δ strain (defective in PI3 kinase complex II that mediates endosomal trafficking), which we previously reported to display hypersensitivity to APOL1 toxicity (Kruzel-Davila et al., 2017a), was less susceptible to the ΔMSALFL constructs (Figure 4A). Because we previously reported diversion of APOL1 from the vacuole to the ER in this yeast strain, we sought to explore whether ΔMSALFL APOL1 localization would be different from full-length APOL1 in *vps38*Δ. The ΔMSALFL APOL1 showed homogeneous distribution in the cytoplasm and as expected was not translocated to the ER, plasma membrane, or vacuole (Figure 4C). This is in sharp contrast to ER localization of APOL1 with an intact and cleavable signal peptide in this yeast strain, implying that APOL1 vacuolar localization is dependent upon the integrity of ER and endosomal trafficking and that the inhibition of ER translocation prevents cleavage of signal peptide and plasma membrane localization. Taken together, these results suggest that the absence of these 6 amino acids inhibits APOL1 translocation to the ER lumen. We hypothesize that these hydrophobic amino acids dictate signal recognition particle (SRP) binding, thereby translocation to the ER and binding to the SRP receptor is impaired in their absence. The disruption of ER luminal translocation attenuates APOL1 toxicity.

### Yeast and Drosophila mutant strains that lack the translocon gene products are protected from APOL1 toxicity

**Figure 5:**
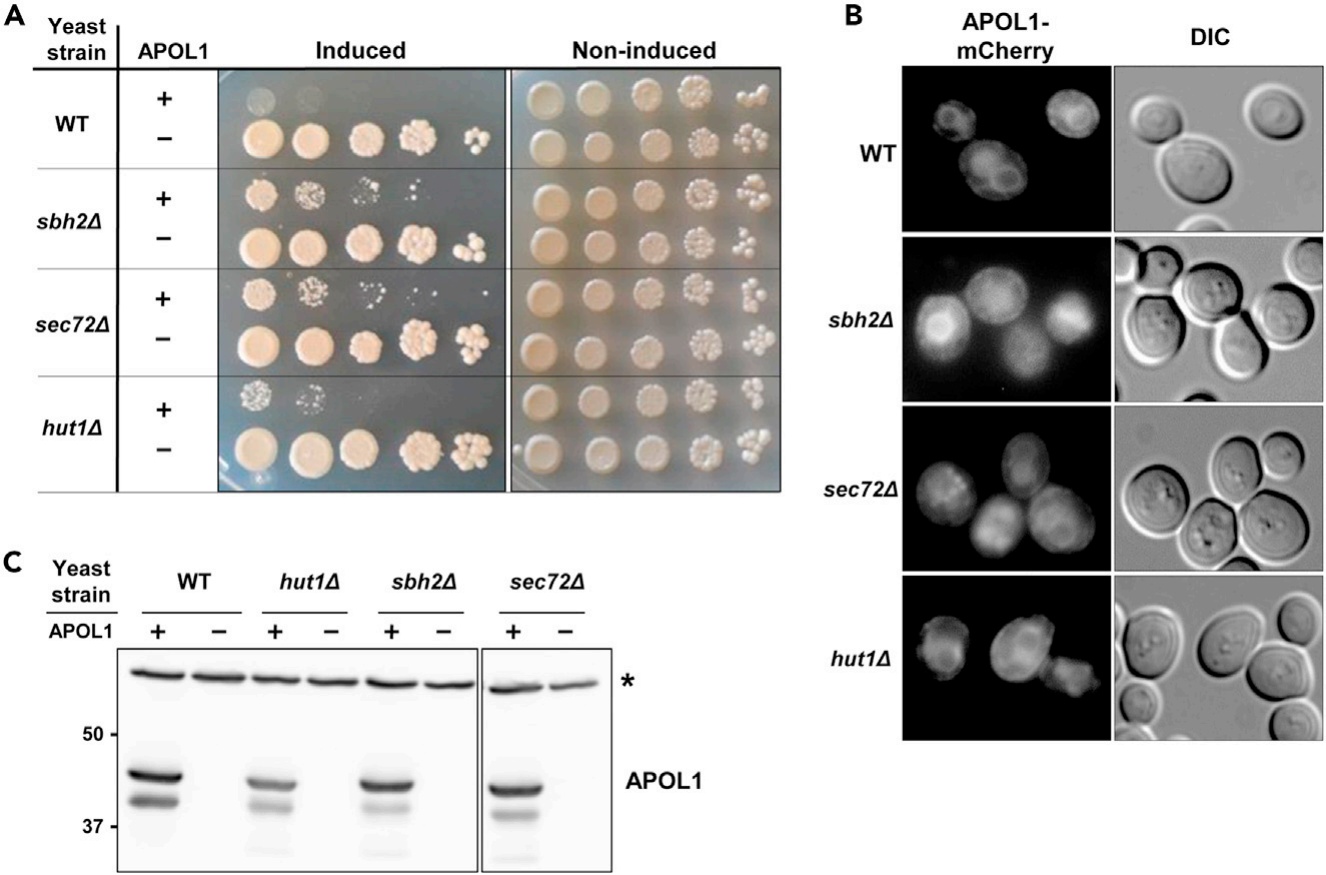
Deletion of key ER genes attenuates APOL1 toxicity and alters its localization Unbiased genetic deletion library screen in yeast expressing APOL1 G2 was used for the discovery of potential proteins that are essential for APOL1 toxicity. The screen identified 3 mutants that were able to attenuate APOL1 toxicity. These proteins are essential for ER translocation (SBH2 and SEC72) or for proteins folding in the ER (HUT1) (A) Drop titration assay shows partial rescue of APOL1 G2 toxicity in *sbh2Δ*, *sec72Δ*, and *hut1Δ* (after 48 h of induction with Galactose). (B) Immunofluorescence of APOL1-mCherry (6h of induction with Galactose) shows reduced ER expression in the mutant strains compared to WT strain. (C) Western blot analysis confirms equal APOL1 expression in all the indicated strains (6h of induction with Galactose). (∗) non-specific protein band that serves as a loading control.

We used a barcoded yeast deletion mutant library (Giaever et al., 2002) in order to identify strains lacking genes which may be involved in a pathway that mediates APOL1 toxicity. As shown in Figure 5A, APOL1 G2 lethality was partially abrogated in strains deleted of genes encoding two key ER translocon proteins, e.g., SBH2 and SEC72 and in a strain deleted of *HUT1*, which is involved in protein folding in the ER (Nakanishi et al., 2001). Immunolocalization of those mutants with *APOL1* G2 fused to mCherry, showed altered cellular staining, compared to the WT strain (Figure 5B), with scant ER staining, supporting the hypothesis that proper translocation of APOL1 to the ER is essential for its toxicity. While the results of these genetic screens cannot be used as a quantitative measure of the contribution of ER luminal localization, nevertheless, they are supportive of the concept that integrity of ER translocation is essential to APOL1 cell injury.

Similarly, we used the fly *HUT1* homolog (*MEIGO)* to investigate whether deletion of this gene would attenuate the lethality of *D. melanogaster* ubiquitously expressing APOL1 (daughterless driver). Flies that are heterozygous for the *MEIGO*^*KG01634*^ allele (an insertional lethal allele) and expressing the toxic APOL1 variants demonstrate improved viability compared to flies expressing these toxic APOL1 variants in the WT background (Table 1), while expressing comparable levels of APOL1 (Figure S6). These results highlight the importance of ER localization in governing APOL1 cellular toxicity.

**Table 1:** Improved viability of APOL1-expressing flies that are heterozygous for *HUT1* homolog (*MEIGO*^*KG01634*^)

	%Survival WT	%Survival MEIGO	p value
G0	97.7	93.5	
G1	6.7	39.6	2.25 × 10^−7^
G2	7.4	33.3	1.92 × 10^−5^

### Inhibition of ER to Golgi trafficking attenuates APOL1 toxicity

**Figure 6:**
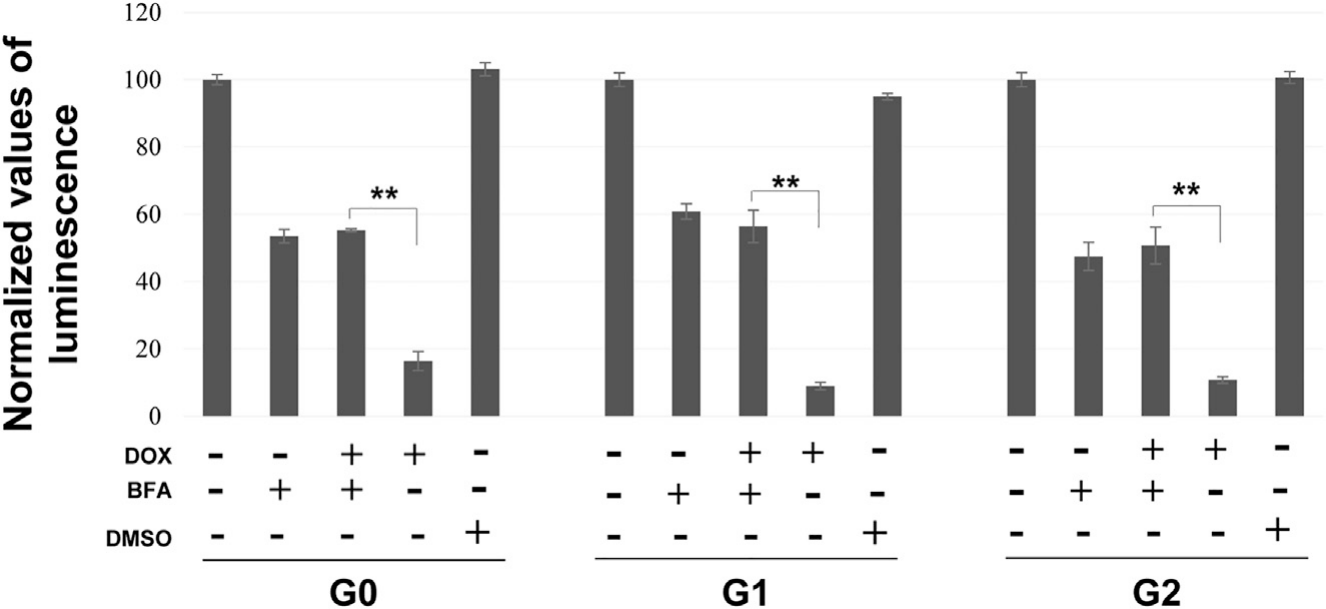
Inhibition of ER to Golgi trafficking attenuates APOL1 toxicity Inducible T-REx 293 cells were induced 16 h with doxycycline (1 ng/mL) for G0, G1, and G2 *APOL1* variants expression (APOL1-flag) in the presence of brefeldin A (10 μg/mL) or DMSO. The viability assay was performed in triplicates and repeated three times, using CellTiter-Glo reagent. Values are means ±SD. T test for statistical significance was conducted for each pair, ∗∗p values <0.001 were considered statistically significant.

In order to decipher whether the ER is the major organelle injured by APOL1 or serves as an essential organelle for proper folding and trafficking of APOL1, we used brefeldin A to block ER to Golgi trafficking in tetracycline-inducible T-REx 293 cells. As shown in Figure 6, brefeldin A partially rescued APOL1 cellular toxicity. This phenomenon was not the result of reduced APOL1 expression by brefeldin A. On the contrary, APOL1 expression was higher in cells treated by brefeldin A (Figure S7). These findings suggest that the ER to Golgi trafficking is necessary for APOL1 cellular injury, rather than direct APOL1 toxicity to the ER.

## Discussion

Recent research has pointed to several potential mechanisms of APOL1 renal risk variant-mediated renal and cellular toxicity. These include mitochondrial dysfunction, potassium efflux, endocytic and autophagic flux impairment, impaired acidification of endolysosomal organelles, pyroptotic cell death, and ER stress (Beckerman et al., 2017; Fu et al., 2017; Granado et al., 2017; Kruzel-Davila et al., 2017a; Ma et al., 2017; Olabisi et al., 2015; Wen et al., 2018). While the initiating event for APOL1 risk variant cell injury remains to be clarified, general blockage of APOL1 at the mRNA level is evolving. Aghajan et al. reported that antisense APOL1 oligonucleotide treatment ameliorates IFN-γ-induced proteinuria in APOL1-transgenic mice (Aghajan et al., 2019). Similar to this approach, we explored the possibility of blocking APOL1 toxicity at a more upstream level, namely, focusing on impaired translocation of APOL1 protein to the ER lumen and from the ER to Golgi.

Pursuant to previously reported work, which demonstrated that exon 4 is essential for APOL1 cellular toxicity (Khatua et al., 2015), we investigated the amino acids needed for ER translocation. The first six amino acids of exon 4—MSALFL—are essential for luminal ER translocation; therefore, signal peptide cleavage is impaired in the absence of these amino acids. The deletion of these amino acids specifically abrogated APOL1 toxicity. As expected, ΔMSALFL APOL1 with defective translocation to the ER lumen retained the signal peptide and was significantly less toxic than APOL1 without deletion of these six amino acids encoded by exon 4, that was localized in the ER lumen. These findings suggest that luminal ER localization is necessary for toxicity, and prevention of translocation into the ER attenuates APOL1 toxicity. Supportive evidence for the importance of APOL1 translocation to the ER stems from a yeast deletion screen, which yielded partial rescue by strains lacking one of the key ER translocon proteins. Although this unbiased screen did not yield other mutants that rescued APOL1 lethality, these mutants only partially improved yeast viability, suggesting that SBH2 and SEC72 are redundant. While the results of these genetic screens cannot be used as a quantitative measure of the contribution of ER luminal localization, nevertheless, they are supportive of the concept that integrity of ER translocation is essential to APOL1 cell injury.

Similarly, flies that ubiquitously expressed APOL1 renal risk variants (G1, G2) and were heterozygous for one of these ER proteins (*HUT1* homolog) demonstrated improved viability compared to flies expressing APOL1 with a WT background, recapitulating the yeast phenotype. In addition, blockade of APOL1 transport from the ER to Golgi attenuated APOL1 toxicity, emphasizing the role of the ER to Golgi trafficking in APOL1 cellular toxicity rather than direct APOL1 toxicity to the ER itself. The essential role of APOL1 trafficking out of the ER is consistent with Giovinazzo et al., which highlighted a proximate role for cation channel activity reaching the plasma membrane in cell injury (Giovinazzo et al., 2020). We conclude that translocation into the ER and trafficking out of the ER are both essential to APOL1 cell injury. Nevertheless, an additional component of ER stress can contribute to APOL1 cellular toxicity, as was recently demonstrated by Wen et al. (Wen et al., 2018).

Cheatham et al. have recently demonstrated that oligonucleotides targeting the 5′ splice site (5′ss) of exon 4 (exon 4 – intron 4–5) can lead to exon 4 skipping in IFN-γ-stimulated AB8/13 podocytes (Cheatham et al., 2018). Given the clinical utility of splice-targeted morpholinos as in Duchenne Muscular Dystrophy (Wood, 2010), we suggest that the morpholino strategy, potentially, could be applied in case of carriers of two APOL1 risk variants to reduce the luminal ER translocation and signal peptide cleavage, thereby leading to a decrease in the extent of cell injury induced by APOL1 risk alleles. Because the heterozygous state for only one APOL1 risk allele is not associated with increased risk for CKD, partial inhibition of APOL1 risk allele expression in those at two-allele genotypic risk might be beneficial. This strategy might be worth pursuing for those at genotypic risk with early indications of kidney disease.

Several studies have demonstrated apparently conflicting results regarding the importance of the APOL1 signal peptide in mediating APOL1 toxicity. These conflicting finding can be reconciled by considering that some studies have used *APOL1* constructs that lack signal peptide and showed that the signal peptide was dispensable for APOL1 cellular toxicity (Granado et al., 2017; Lan et al., 2015a; Nichols et al., 2015; Thomson et al., 2014), while other studies used *APOL1* constructs that harbored non-cleavable signal peptide (Khatua et al., 2015). Granado et al., and recently Muller et al., used GFP-*APOL1* constructs that lack the signal peptide and were still expressed in the ER while retaining the toxicity potential (Granado et al., 2017; Muller et al., 2021). Muller et al. have fused N glycosylation tag (GT, Glyco-tag) that only becomes glycosylated in the ER lumen and elegantly demonstrated that APOL1 protein in which the SP (aa1– 28) was replaced by an N-terminal GFP-tag was facing the cytoplasmic side of the ER but still retained APOL1 toxicity (Muller et al., 2021). Their findings do not reconcile with our conclusion of reduced toxicity of APOL1 that faces the cytoplasmic face of the ER compared to isoforms which insert into the ER lumen. These discrepancies might stem from the isoform-dependent duality of the mechanism for cell injury suggested in Muller et al. and Wakashin et al. (Muller et al., 2021; Wakashin et al., 2020), or possibly the GFP-APOL1 construct used compared to our artificial ΔMSALFL APOL1, that might be more toxic to cells. Notably, we used the African background (E150, I228, K255) in all constructs, therefore the attenuated ΔMSALFL APOL1 toxicity cannot be explained by a different APOL1 haplotype (Lannon et al., 2019). Therefore, we conclude that the diversion from lumen of the ER and localization at the cytoplasmic face of the ER leads to retention of APOL1 signal peptide and attenuates APOL1 toxicity. We suggest that inhibition of APOL1 translocation into the lumen of the ER may serve as a potential therapeutic target that might mitigate APOL1 toxicity and the risk for kidney disease in individuals with the two APOL1 risk allele genotype. Further studies are needed to explore this therapeutic avenue.

### Limitation of the study

This study shows reduced toxicity of ΔMSALFL when exogenously overexpressed in the model systems. This method does not reflect the endogenous cellular levels of APOL1, which may have an effect on its localization.

We did not reconcile or address potential mechanisms for cell injury reportedly induced by a splice isoform (VB3) lacking the exon 4 (Wakashin et al., 2020).

We did not explore the therapeutic potential of ΔMSALFL in a bacterial artificial chromosome (BAC) transgenic murine model harboring either the wild-type (G0), G1 or G2 forms of human APOL1 (McCarthy et al., 2021).

## STAR★Methods

### Key resources table

**Table:** 

REAGENT or RESOURCE	SOURCE	IDENTIFIER
**Antibodies**		

Mouse monoclonal anti c-Myc (9E10)	Santa Cruz Biotechnology	Cat# sc-40; RRID:AB_627268
Mouse monoclonal ANTI-FLAG (M2)	Sigma-Aldrich	Cat# F1804; RRID:AB_262044
Mouse monoclonal anti-Calnexin (TO-5)	Santa Cruz Biotechnology	Cat# sc-80645; RRID:AB_1119919
Mouse monoclonal anti-PDI (RL90), Alexa Fluor 488	Thermo Fisher Scientific (Invitrogen)	Cat# MA3019A488; RRID:AB_2633336
Rabbit polyclonal anti-APOL1	Sigma-Aldrich	Cat# HPA018885; RRID:AB_1844953
Rabbit monoclonal anti-APOL1 (5.17D12)	Genentech, Scales et al. (2020)	Lot# PUR136588
Rabbit polyclonal anti-GAPDH	Santa Cruz Biotechnology	Cat# sc-25778; RRID:AB_10167668
Mouse monoclonal anti-alpha-Tubulin	Sigma-Aldrich	Cat# T5168; RRID:AB_477579
Goat polyclonal anti-Rabbit IgG (H+L) Horseradish Peroxidase conjugated	Jackson ImmunoResearch Labs	Cat# 111-035-144; RRID:AB_2307391
Goat polyclonal anti-Mouse IgG (H+L) Horseradish Peroxidase conjugated	Jackson ImmunoResearch Labs	Cat# 115-035-166; RRID:AB_2338511
Donkey polyclonal anti-Rabbit IgG (H+L) Cyanine Cy3 conjugated	Jackson ImmunoResearch Labs	Cat# 711-165-152; RRID:AB_2307443
Donkey polyclonal anti-Mouse IgG (H+L) Alexa Fluor 488 conjugated	Jackson ImmunoResearch Labs	Cat# 715-545-150; RRID:AB_2340846
Rabbit polyclonal anti-Calnexin	Enzo Life Sciences	Cat# ADI-SPA-860; RRID:AB_10616095
Rabbit polyclonal anti-Human SDHB (succinate dehydrogenase complex iron sulfur subunit B)	LSBio	Cat# LS-C497529

**Chemicals, peptides, and recombinant proteins**		

Digitonin	Sigma-Aldrich	Cat# D141
Saponin	Sigma-Aldrich	Cat# 47036

**Critical commercial assays**		

CellTiter-Glo Luminescent Cell Viability assay	Promega	Cat# G7571
Pierce LDH Cytotoxicity Assay Kit	Thermo Scientific	Cat# 88953

**Experimental models: Cell lines**		

Human: tetracycline-inducible T-REx 293 APOL1 G0	This paper	N/A
Human: tetracycline-inducible T-REx 293 APOL1 G1	This paper	N/A
Human: tetracycline-inducible T-REx 293 APOL1 G2	This paper	N/A
Human: tetracycline-inducible T-REx 293 APOL1 G0 ΔMSALFL		N/A
Human: tetracycline-inducible T-REx 293 APOL1 G1 ΔMSALFL	This paper	N/A
Human: tetracycline-inducible T-REx 293 APOL1 G2 ΔMSALFL	This paper	N/A
Human: HEK-293T	ATCC	ATCC #CRL-3216; RRID:CVCL_0063
Human: HEK-293	ATCC	ATCC #CRL-1573, RRID:CVCL_0045

**Experimental models: Organisms/strains**		

*S. cerevisiae: WT* BY4741; Mat a; his3Δ1, leu2Δ0, met15Δ0, ura3Δ0	Euroscarf	Y00000
*S. cerevisiae: vps28 Δ;* BY4741; vps28:: kanMX4	Euroscarf	Y02763
*S. cerevisiae: hut1 Δ;* BY4741; hut1::kanMX4	Euroscarf	Y01048
*S. cerevisiae: sbh2;* BY4741; sbh2::kanMX4	Euroscarf	Y00151
*S. cerevisiae: sec72;* BY4741; sec72::kanMX4	Euroscarf	Y05202
*D. melanogaster*: *w*^*1118*^	Laboratory of Adi Salzberg	N/A
*D. melanogaster*: *da-GAL4*	Laboratory of Adi Salzberg	N/A
*D. melanogaster*: *MEIGO*^*KG01634*^	Bloomington Drosophila Stock Center	Cat# 13460
*D. melanogaster*: *UAS-APOL1 G0*	Kruzel-Davila et al. (2017a)	N/A
*D. melanogaster*: *UAS-APOL1 G1*	Kruzel-Davila et al. (2017a)	N/A
*D. melanogaster*: *UAS-APOL1 G2*	Kruzel-Davila et al. (2017a)	N/A
*D. melanogaster*: *UAS-APOL1 G0; MEIGO*^*KG01634*^	This paper	N/A
*D. melanogaster*: *UAS-APOL1 G1; MEIGO*^*KG01634*^	This paper	N/A
*D. melanogaster*: *UAS-APOL1 G2; MEIGO*^*KG01634*^	This paper	N/A

**Oligonucleotides**		

Primer for APOL1 isoform B cloning to pCMV6-XL5 (forward, EcoRI site underlined): TCGGAATTCGCCATGAGATTCAAAAGCCAC	This paper	N/A
Primer for APOL1 isoform B cloning to pCMV6-XL5 (reverse, XbaI site underlined): AGATTCTAGATCACAGTTCTTGGTCCGCCT	This paper	N/A
Primer for APOL1 isoform C cloning to pCMV6-XL5 (forward): GAGTCTCTGTCCTCTGCATCTGGGTG CAACAAAACGTTCCAAGTGGG	This paper	N/A
Primer for APOL1 isoform C cloning to pCMV6-XL5 (reverse): CCCACTTGGAACGTTTTGTTGCACCC AGATGCAGAGGACAGAGACTC	This paper	N/A
Primer for Flag-Apol1-Myc cloning to pFlag-CMV-2 (forward, NotI site underlined): ATGCGCGGCCGCGATGAGATTCAAAAGCCACAC	This paper	N/A
Primer for Flag-Apol1-Myc cloning to pFlag-CMV-2 (reverse, BamHI site underlined): GCAGGGATCCTCACAGGTCCTCCTCTGAGATC	This paper	N/A
Primer for replacing *APOL1-*mCherry with ΔMSALFL in p426-GAL1 (forward, SpeI site underlined): AGCTACTAGTAT GAGATTCAAAAGCCACAC	This paper	N/A
Primer for replacing *APOL1-*mCherry with ΔMSALFL in p426-GAL1 (reverse, BamHI site underlined): AGCTGGATCCTTCAGTTCTTGGTCCGCCTGCAG	This paper	N/A

**Recombinant DNA**		

cDNA: *APOL1* isoform A		NM_003661
cDNA*: APOL1* isoform B1	Geneart, Life Technologies	NM_145343
cDNA: *APOL1* isoform C		NM_001136541
Plasmid: pCMV6-XL5	OriGene	Cat# PCMV6XL5
Plasmid: pFlag-CMV-2	Sigma	Cat# E7033
Plasmid: pcDNA4/TO	Thermo Fisher Scientific (Invitrogen)	Cat# V102020
Plasmid: p426-GAL1 (2 μ; URA3) G0	Kruzel-Davila et al. (2017a)	N/A
Plasmid: p426-GAL1 (2 μ; URA3) G1	Kruzel-Davila et al. (2017a)	N/A
Plasmid: p426-GAL1 (2 μ; URA3) G2	Kruzel-Davila et al. (2017a)	N/A
Plasmid: p426-GAL1 (2 μ; URA3) G0 ΔMSALFL	This paper	N/A
Plasmid: p426-GAL1 (2 μ; URA3) G1 ΔMSALFL	This paper	N/A
Plasmid: p426-GAL1 (2 μ; URA3) G2 ΔMSALFL	This paper	N/A

### Resource availability

#### Lead contact

Further information and requests for resources and reagents should be directed to and will be fulfilled by the lead contact, Etty Kruzel-Davila, Nephrology Department, Galilee Medical Center, Nahariya, Israel. Email: ETTYK@gmc.gov.il, etty.kruzel@gmail.com.

#### Materials availability

Plasmids and flies strains generated in this study are available from the lead contact upon request.

### Experimental model and subject details

#### Cell cultures

HEK293T and HEK293 cells were originally obtained from the American Type Culture Collection (ATCC, Manassas, VA, #CRL-3216, #CRL-1573, respectively). The cells were cultivated in high glucose Dulbecco’s Modified Eagle’s Medium (DMEM, Invitrogen) supplemented with 10% heat-inactivated fetal bovine serum (Invitrogen) and gentamicin (50 μg/ml, Invitrogen). HEK293 cell line was cultivated in standard DMEM medium (Thermo Fisher Scientific) supplemented with 10% FCS and 1% antibiotics (penicillin/streptomycin). Transient transfection was performed with PolyJet as described according to the manufacturer’s instructions (Signagen Laboratories).

For the generation of tetracycline-inducible APOL1 T-REx 293 cells, *APOL1* G0, G1 and G2, conjugated to Flag-tag at the C-terminus, were cloned into pcDNA4/TO (Invitrogen) using XhoI and NotI or BamHI and XhoI restriction sites, respectively, and transfected to T-REx 293 cells (Invitrogen) using PolyJet reagent. Inducible T-REx 293 cell line was cultivated like HEK293 with the addition of Zeocin and Blasticidin S (200 μg/ml and 5 μg/ml, respectively, InvivoGen). Expression of *APOL1* constructs was induced with doxycycline (Sigma), a tetracycline analog.

#### Yeast strains and media

The *S. cerevisiae* strains used in this study are listed in Table S1. The strains were grown at 30°C in standard yeast extract/peptone/dextrose (1% yeast extract, 2% peptone, and 2% dextrose), complete yeast nitrogen base medium (1.5 g yeast nitrogen base per/ L, 5 g ammonium sulfate per / L, 2% glucose or galactose, and 0.1 g/L each amino acid with the appropriate amino acids removed as required for plasmid selection), or minimal medium (1.5 g yeast nitrogen base per / L, 5 g ammonium sulfate per / L, 2% glucose or galactose, and 0.1 g/L essential amino acids).

#### Fly strains

The following strains were used in this study (described in FlyBase http://flybase.org, Gramates et al., 2017(Gramates et al., 2017)): *w*^*1118*^served as a wild type control, *da*-GAL4 was used to drive transgene expression ubiquitously as a model for systemic APOL1 expression. For the generation of APOL1 transgenic flies, APOL1 G0, G1, G2 and C–terminal truncated construct that lacks the serum resistance–associated (SRA) interacting domain, were cloned into the pUASTattB vector at NotI and BglII (Bischof et al., 2007), and transgenic strains were generated by ΦC31 integrase–based transgenesis. All transgenes were inserted into the attP2 chromosomal landing site (Genetic Services Inc., Cambridge, MA and BestGene Inc., Chino Hills, CA) (described previously in (Kruzel-Davila et al., 2017a). The *MEIGO*^*KG01634*^ allele (Bloomington Drosophila Stock Center: #13460) is an insertional lethal allele generated as part of the Gene Disruption Project. Recombinant chromosomes carrying the *UAS*-*APOL1* G0, G1, G2 together with the *MEIGO*^*KG01634*^ mutation were generated by meiotic recombination. Flies were grown on standard corn meal-yeast-molasses fly food at 24°C. Gal4/UAS crosses were incubated at 29°C. Newly eclosed flies from each cross were collected on the same day and transferred to fresh vials for analysis. The presented values are means of three independent experiments, each performed with three different clones for each variant.

### Method details

#### Plasmids

Figure S1 provides a schematic representation of the known APOL1 splice isoforms. *APOL1* cDNA corresponding to splice isoform A (GenBank: NM_003661) was consructed in pCMV6-XL5 vector (OriGene). *APOL1* isoform B1 (GenBank: NM_145343, Geneart, Life Technologies) was PCR-amplified from a provided vector using primers containing EcoRI and XbaI restriction sites (underlined): 5′-TCGGAATTCGCCATGAGATTCAAAAGCCAC-3′ (forward EcoRI primer) and 5′- AGATTCTAGATCACAGTTCTTGGTCCGCCT-3′ (reverse XbaI primer). *APOL1* isoform C (GenBank: NM_001136541) was generated by deletion of 54 nucleotides corresponding to the full exon 4 of *APOL1* isoform A using a set of two complementary primers: 5′-GAGTCTCTGTCCTCTGCATCTGGGTGCAACAAAACGTTCCAAGTGGG-3′ and 5′-CCCACTTGGAACGTTTTGTTGCACCCAGATGCAGAGGACAGAGAC TC-3′. The PCR product corresponding to full-length *APOL1* cDNA was cloned into EcoRI and XbaI sites of pCMV6-XL5. The open reading frame of *APOL1* genes expressed from pCMV6-XL5 was modified by PCR with Myc epitope (Khatua et al., 2015). *APOL1* deletion mutants were created using the QuickChange II site-directed mutagenesis kit (Stratagene). Integrity of *APOL1* constructs was confirmed by DNA sequencing. For the Flag-*APOL1*-Myc constructs, the cDNA was amplified and cloned into pFlag-CMV-2 plasmid (Sigma) using primers containing NotI and BamHI restriction sites (underlined): 5'-ATGCGCGGCCGCGATGAGATTCAAAAGCCACAC (forward NotI primer) and 3'-GCAGGGATCCTCACAGGTCCTCCTCTGAGATC (reverse BamHI primer).

For expression in yeast under the Gal1 promoter, *APOL1* G0 (isoform B1: 414 amino acids; transcript variant 2; GenBank: NM_145343), G1, and G2 synthetic cDNAs (purchased from Hy-labs) were cloned to high–copy p426-GAL1 (2 μ; URA3) vector using BamHI and EcoRI restriction sites. The *APOL1*-mCherry constructs were cloned by introducing the mCherry at the C terminus by fusion PCR (described previously in (Kruzel-Davila et al., 2017a)). For replacing APOL1*-*mCherry with ΔMSALFL we used primers containing SpeI and BamHI restriction sites (underlined): 5'-AGCTACTAGTATGAGATTCAAAAGCCACAC (forward SpeI primer) and 3'- AGCTGGATCCTTCAGTTCTTGGTCCGCCTGCAG (reverse BamHI primer).

All constructs used in this study are based on the African genetic background E150, I228, K255 (Lannon et al., 2019).

#### Western Blot

Lysate protein samples were separated by SDS-PAGE and blotted onto Nitrocellulose Membranes (Amersham). The membranes were blocked with 5% nonfat dry milk (Santa Cruz) in Tris buffered saline with Tween 20 and incubated with primary antibodies as indicated below. The membranes were then incubated with the appropriate secondary antibodies (as indicated below). After extensive washing in Tris-buffered saline with Tween 20, the proteins were visualized using chemiluminescence reagents.

#### Antibodies

Anti-Myc (sc-40, Santa Cruz), anti-Flag (F1804, Sigma), anti-C-terminal Calnexin (1:50, sc-80645, Santa Cruz), anti-PDI-A488 (9 μg/ml, #MA3019A488, Invitrogen), anti-APOL1 (HPA018885, Sigma), anti-APOL1 5.17D12 (1 μg/ml, kindly provided by Genentech), anti-GAPDH (sc-25778, Santa Cruz), anti-Tubulin (T5168, Sigma). Anti-rabbit HRP (1:10,000; 111-035-144, Jackson) and anti-mouse HRP (1:10,000; 115-035-166, Jackson) were used as secondary antibodies for WB. Cy3-labeled anti-rabbit (1:350, #711-165-152, Jackson) and Alexa488-labeled anti-mouse (1:350, #715-545-150, Jackson) were used as secondary antibodies for IF.

For fractionation assay: anti- APOL1 (HPA018885, Sigma), anti-Calnexin (ADI-SPA-860, ENZO), anti-Tubulin (T5168, Sigma), anti-SDHB (LS-C497529, LSBio) were used.

#### Immunofluorescence

Inducible T-REx 293 cells containing full length and ΔMSALFL *APOL1* variants were plated on coverslips coated with poly-L-lysine at density of 180 K cells per well. After 24 hours, cells were induced with 20 ng/ml doxycycline for 130 min and fixed for 10 min in 4% PFA. Cells were permeabilized 20 min with saponin buffer (0.4 % saponin, 1% BSA, 2% FBS in PBS) or 4 min with 16 μM digitonin in KHM buffer (110 mM potassium acetate, 20 mM HEPES pH 7.4, 2 mM MgCl_2_). Then, cells were subjected to immunofluorescence staining with APOL1 5.17D12 (1 μg/ml, kindly provided by Genentech) (Gupta et al., 2020; Scales et al., 2020), C-terminal Calnexin (CNX), as a control for cytoplasmic orientation (1:50, sc-80645, Santa Cruz) or PDI-A488, as a control for luminal ER localization (9 μg/ml, #MA3019A488, Invitrogen) antibodies for 90 min at 37°C. The cells were then washed with PBS or KHM buffer three times for 5 min each, and incubated with Cy3-labeled anti-rabbit or Alexa488-labeled anti-mouse secondary antibodies (1:350) at 37°C for 1 h. After washes, stained cells were mounted in Antifade Mounting Medium with DAPI (H-1200, Vectashield). Images were acquired using Confocal LSM 700 Inverted Microscope at x63 magnification.

#### Cellular fractionation

Ten 15-cm plates of T-REx 293 cells containing *APOL1* flag-G1 / G1Δ-myc were induced for 3 hours with 20 ng/ml doxycycline, then were harvested by trypsinization and manually homogenized in 10 ml buffer (225 mM mannitol, 75 mM sucrose, 30 mM Tris-HCl, 0.1 mM EGTA, pH7.4) with about 100 strokes of a glass conical homogenizer, followed by sonication (8 sec x 3 times, wheel set to 3.5 in a Misonix Sonicator XL2020 Ultrasonic Liquid Processor). Cells were then fractionated by differential centrifugation according to Wieckowski et al. (Wieckowski et al., 2009) as follows. Cell homogenates were first centrifuged to get total lysate (600 ×g, 5 min, 3 times), then the crude mitochondria was pelleted by centrifugation (7,000 ×g, 10 min, followed by two washes and 10,000 ×g, 10 min centrifugation). Lysosomes and plasma membranes were pelleted from the first mitochondrial supernatant (20,000 ×g, 30 min), whereas the ER was separated from cytosolic fraction by ultra-centrifugation at 100,000 ×g for 60 min. All pellets were resuspended in 250 mM mannitol, 5 mM HEPES pH 7.4, 0.5 mM EGTA, and 30 ug total protein samples from each fraction were loaded per lane of Tris-Glycine gel and probed with representative antibodies.

#### Cell viability assay

Inducible T-REx 293 cells containing full length and ΔMSALFL *APOL1* variants were seeded at density of 20 K cells per well in 96-well plate. After 24 hours of growth, cells were treated with doxycycline and brefeldin A (Sigma) for 16 hours and cell viability was measured using CellTiter-Glo Luminescent Cell Viability assay (Promega) according to the manufacture instructions. Cells were grown at least three days in Tet-free medium before doxycycline induction.

#### Lactate dehydrogenase (LDH) toxicity assay

HEK293T cells, cultured on six-well plates, were transfected with 1 μg control pCMV6-XL5 empty vector (OriGene) and 1 μg of indicated *APOL1* constructs per well using Polyethylenimine (PEI, 25,000 mol wt, Polysciences). After 5 h, the media were replaced with DMEM containing 2% FCS. After 24 h, culture media were harvested and centrifuged for 15 min at low speed to remove cell debris, and LDH activity was quantitated using a Pierce™ LDH Cytotoxicity Assay Kit (Thermo Scientific) following the manufacturer's protocol. Enzyme activity of LDH was assayed quantitatively by measurement of absorbance at 490 nm using a microplate reader. Cytotoxicity was expressed as a percentage of LDH released in experimental samples (subtracted for LDH present in 2% FCS culture medium) relative to LDH released by totally lysed cells (set at 100%).

#### Drop titration assay

p426-GAL1 (2 μ; URA3) plasmids containing the human full length and ΔMSALFL *APOL1* variants under the yeast GAL1 promoter were transformed into yeast strains by using the standard lithium acetate method. Serial fivefold dilutions of cell suspensions transformed with the indicated plasmid spotted on plates containing glucose (non-induced conditions) or galactose (induced conditions) were conducted. The assay was conducted after 48 hours of induction.

#### Yeast deletion mutant library

The barcoded library (Giaever et al., 2002) was transformed with *APOL1*-G2 plasmid. Transformed cells were diluted and plated on induction plates (–URA +2% Galactose pH 5.3), colonies that were able to grow were isolated and barcodes were identified by PCR. Candidate strains were ordered from Euroscarf (Table S1), transformed with *APOL1*-G2 or empty vector (EV) and were verified by drop titration assay.

### Quantification and statistical analysis

Statistical analysis was conducted using pairwise t-tests for comparisons of two groups. P values < 0.05 (marked with ∗), P<0.001 (marked with ∗∗) were considered statistically significant.

## Data Availability

• Original WB, drop titration assays and microscopy images, and cell viability raw data measurements will be shared by the lead contact upon request. • This paper does not report original code. • Any additional information required to reanalyze the data reported in this paper is available from the lead contact upon request.
