# Supplementary material for: Endoplasmic reticulum-translocation is essential for APOL1 cellular toxicity
Source: iScience. 2021 Dec 31;25(1):103717. doi: 10.1016/j.isci.2021.103717 (PMC8762391; doi:10.1016/j.isci.2021.103717)
Supplement: Document S1. Figures S1–S7 and Table S1 [file mmc1.pdf]

iScience, Volume *25*

Supplemental information

Endoplasmic reticulum-translocation

is essential for APOL1 cellular toxicity

Etty Kruzel-Davila, Ira Bavli-Kertselli, Ayala Ofir, Amber M. Cheatham, Revital Shemer, Eid Zaknoun, Sergiy Chornyy, Orly Tabachnikov, Shamara E. Davis, Atanu K. Khatua, Karl Skorecki, and Waldemar Popik

Supplemental Data

Table S1: Yeast strains that were used in the study, Related to Figures 4 and 5.

Name	Relevant genotype	Source
WT	BY4741; Mat a; his3Δ1, leu2Δ0, met15Δ0, ura3Δ0	Euroscarf
vps28Δ	BY4741; vps28:: kanMX4	Euroscarf
hut1 Δ	BY4741; hut1::kanMX4	Euroscarf
sbh2	BY4741; sbh2::kanMX4	Euroscarf
sec72	BY4741; sec72::kanMX4	Euroscarf

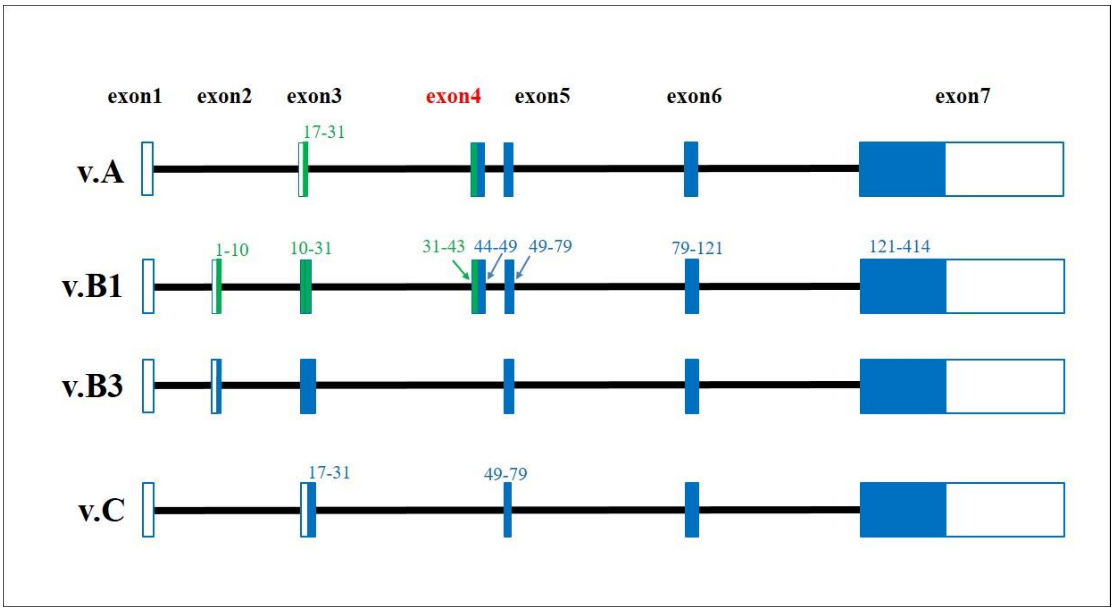

Figure S1. Schematic of *APOL1* splice transcript isoforms, Related to Figure 1. The major *APOL1* splice isoforms as cataloged in the National Center for Biotechnology information database. Isoform A is encoded by transcript isoform 1 (NM_ 003661) and contains exons1 and 3 to7 (protein composed of 398 amino acids); isoform B1 is encoded by transcript isoform 2 (NM_ 145343) and harbors exons 1 to 7 (414 amino acids); isoform B3 is similar to isoform B1 but lacks exon 4 (396 amino acids); isoform C is encoded by transcript isoform 4 (NM_ 001136541) and contains exons 1, 3, and 5 to 7 (380 amino acids). Untranslated exons or parts of exons are indicated by white boxes; exons encoding a putative signal peptide are shown in green, and translated exons are shown in blue. Reprinted with permission from Khatua et al., 2015 and Kruzel-Davila et al., 2017b.

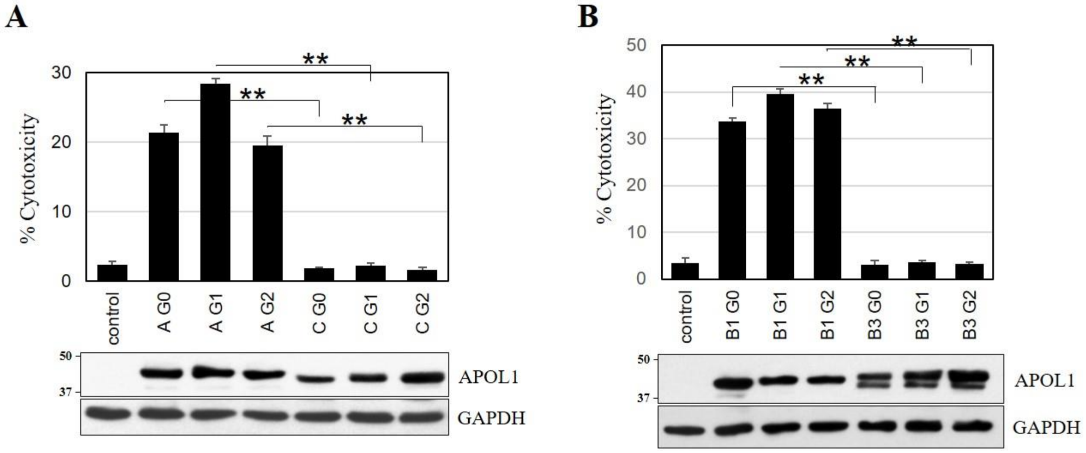

Figure S2. *APOL1* splice isoform toxicity measured by LDH assay, Related to Figure 1. (A) Isoforms A and C. **(B)** Isoforms B1 and B3. Values are means ±SD of 3 independent experiments. T-test for statistical significance was conducted for each pair, P values **P<0.001 were considered statistically significant.

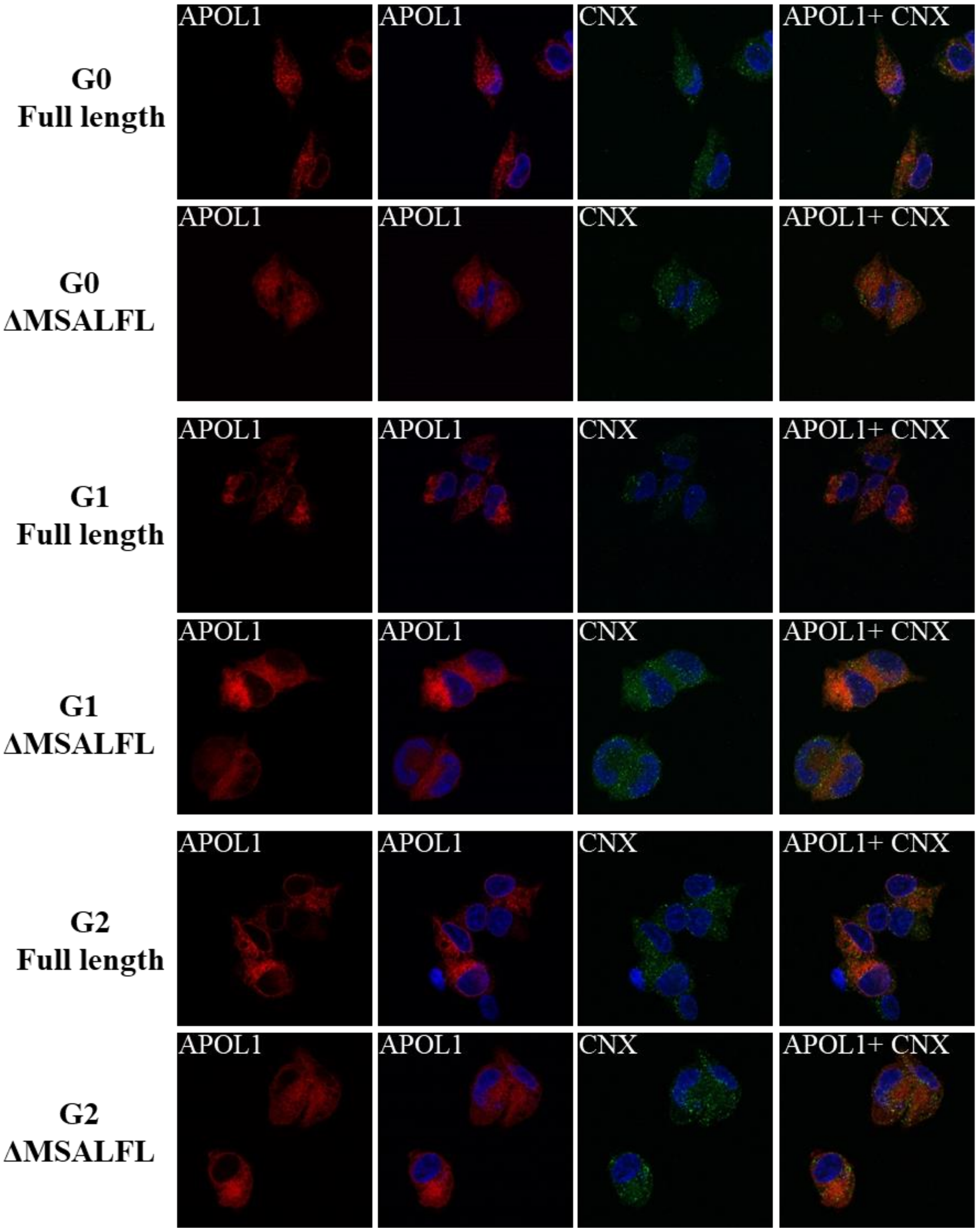

Figure S3. APOL1 variants are localized at the ER, Related to Figure 3. Inducible T-REx 293 cells were induced for APOL1 (flag-APOL1-myc) expression by doxycycline (20 ng/ml) and permeabilized with saponin. All APOL1 variants and ΔMSALFL constructs demonstrate partial colocalization with calnexin (CNX, C-terminal part).

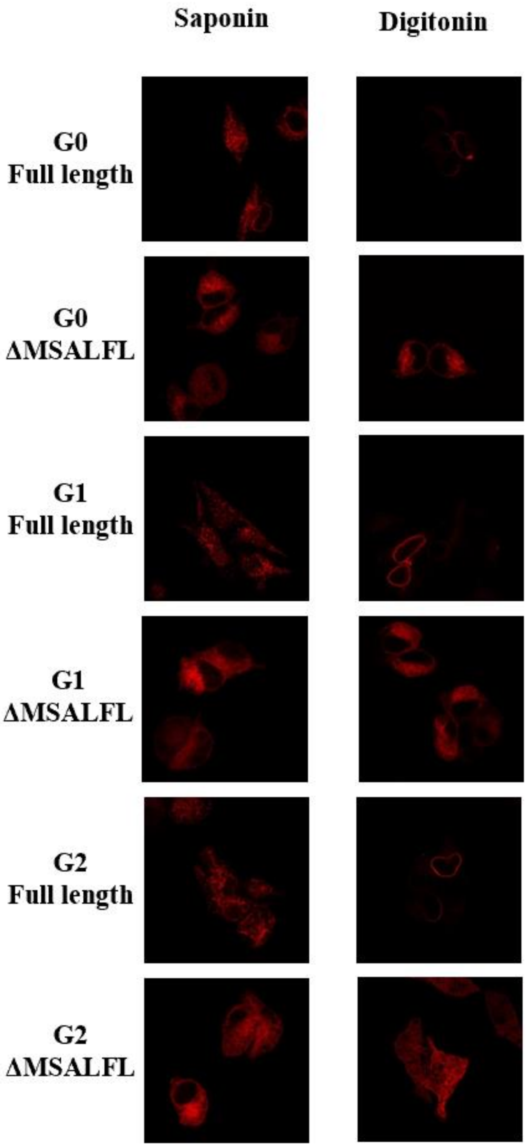

Figure S4. ΔMSALFL constructs localize at the cytoplasmic face of the ER as opposed to the luminal ER localization of full length APOL1 constructs, Related to Figure 3. Inducible T-REx 293 cells were induced for APOL11 (flag- APOL1-myc) expression by doxycycline (20 ng/ml) and permeabilized with saponin (left) or digitonin (right). The reticular ER pattern persists in digitonin (permeabilizes plasma membrane only) permeabilized cells expressing the ΔMSALFL constructs but not full length APOL1 G0, G1, G2, indicating ΔMSALFL constructs localize at the cytoplasmic face of the ER, while full length APOL1 is localized within the ER, thereby not accessiable to staining after digitonin permeabilization as opposed to saponin permeabilization.

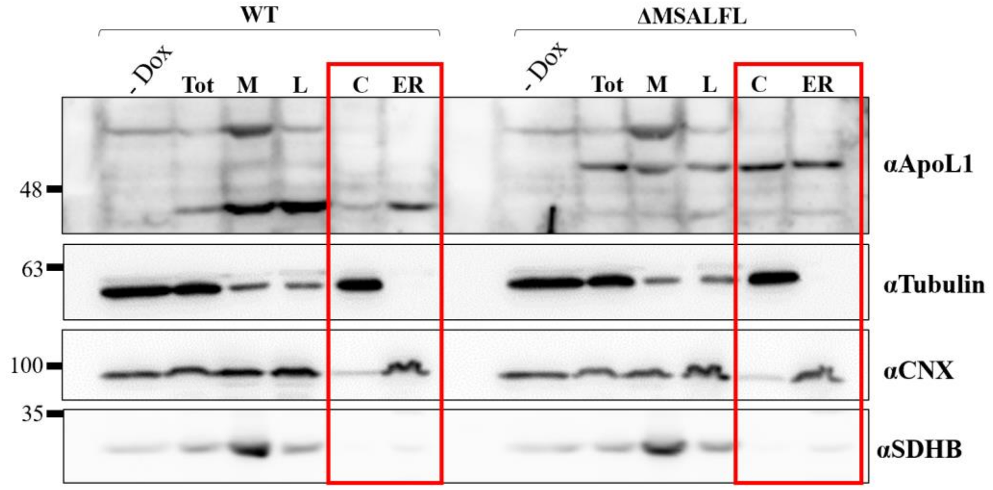

Figure S5. **WT and ΔMSALFL APOL1 isoforms are located in different fractions of cellular organelles, Related to Figure 3.** Western blot analysis of subcellular localization from inducible T-REx 293 cells shows different cellular localization for non-deleted and ΔMSALFL APOL1 constructs. WT APOL1 is found only in the ER fraction whereas ΔMSALFL APOL1 is expressed in the ER as well as the cytosol fraction due to its localization at the cytoplasmic face of the ER. Cells containing APOL1 G1 WT or ΔMSALFL constructs were induced for 3 hours with 20 ng/ml doxycycline and subjected to cellular fractionation. 30 µg of proteins were loaded per each lane. Cellular compartments were verified by using different markers: alpha-Tubulin for cytosol (C), Calnexin (CNX) for Endoplasmatic Reticulum (ER) and succinate dehydrogenase complex iron sulfur subunit B (SDHB) for mitochondria (M). Total protein was loaded as unfractionated control (Tot).

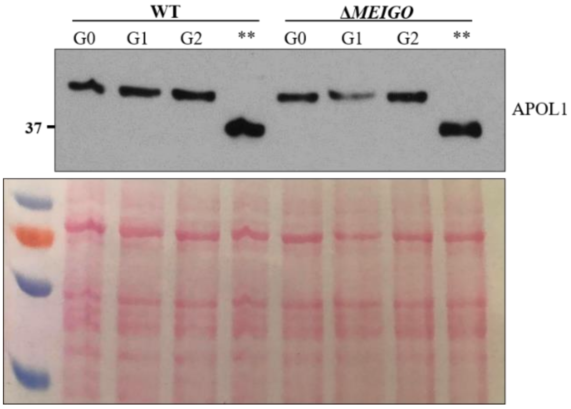

Figure S6. APOL1 expression levels in *MEIGO* deletion flies are similar to WT strain, Related to Table 1. Western Blot of flies expressing APOL1 in WT and in *MEIGO* deletion strains demonstrate equal amount of protein levels. The Ponceau staining demonstrates equivalent protein loading. (**) APOL1 C terminal truncated- this construct was used as a control, given our previous work that demonstrated that this construct is innocuous to flies.(Kruzel-Davila et al., 2017a)

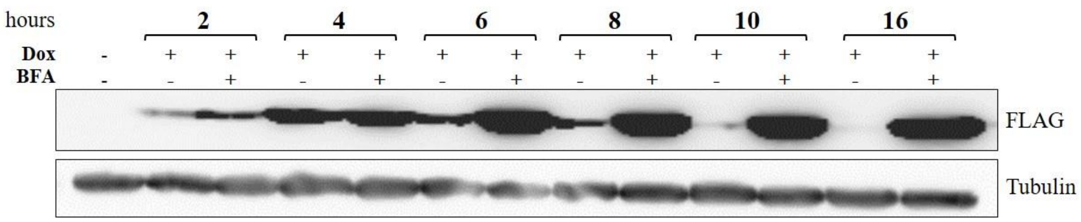

Figure S7. Amelioration of cell viability by brefeldin A is not mediated by reduced APOL1 expression, Related to Figure 6. Inducible T-REx 293 cells were induced as indicated with doxycycline (1 ng/ml) for G1 APOL1-flag with or without brefeldin A (10 µg/ml). APOL1 expression is preserved with brefeldin A, whereas decreased expression was demonstrated after 6 hours without brefeldin A, reflecting reduced viability of APOL1 expressing cells.
